# Phylogenetic relationship of *Paramignya trimera* and its relatives: an evidence for the wide sexual compatibility

**DOI:** 10.1038/s41598-020-78448-2

**Published:** 2020-12-10

**Authors:** Thi Cam Mien Phi, Hoang Ha Chu, Ngoc Trieu Le, Duc Bach Nguyen

**Affiliations:** 1 Faculty of Biotechnology, Vietnam National University of Agriculture, Ngo Xuan Quang, Trau Quy, Hanoi Vietnam; 2 Institute of Biotechnology, Vietnam Academy of Science and Technology, 18 Hoang Quoc Viet, Cau Giay, Hanoi, Vietnam; 3 Faculty of Biology, Dalat University, No1 Phu Dong Thien Vuong, Dalat, Lam Dong Vietnam

**Keywords:** Ecology, Evolution, Molecular biology, Plant sciences

## Abstract

The genus *Paramignya* (Rutaceae) comprises about 30 species typically distributing in tropical Asia. Like other genera of the family Rutaceae, the significant variation in the morphology of *Paramignya* species makes the taxonomic study and accurate identification become difficult. In Vietnam, *Paramignya* species have been mostly found in Khanh Hoa and Lam Dong provinces and used as traditional medicines. Recently, *Paramignya trimera*, a species of the genus *Paramignya* with local name “Xao tam phan” has been drawn attention and intensively exploited to treat liver diseases and cancers. However, the significant variations in the morphology and different local names of *P. trimera* have caused confusion and difficulty in the accurate identification and application of this plant for medicine. In this study, the combination of both morphological and DNA sequence data has effectively supported the taxonomic identification of *P. trimera* and some relatives collected in Khanh Hoa and Lam Dong provinces. The comparison of the morphology and analysis of the phylogenetic trees suggested that there was a significant variation of *P. trimera*. In addition, some accessions of *P. trimera* with morphological characteristics similar and *Atalantia buxifolia* were likely the intergeneric hybrids between the two species. Analysis of genetic variation, interspecific and intraspecific distances using ITS, matK and rbcL sequences shown that *P. trimera* was closely related to *A. buxifolia*, *Severinia monophylla* and *Luvunga scandens*. In addition, matK sequences represented as the effective candidate DNA barcode to identify and distinguish *Paramignya* species from others of the family Rutaceae.

## Introduction

Genus *Paramignya* belongs to the subtribe Triphasiinae, tribe Citreae, subfamily Aurantioideae and family Rutaceae^1,2^. *Paramignya* species are woody shrub plants widely distributing in the tropical regions such as Southern Vietnam, Philippines, Thailand, Malaysia, Java-Indonesia, Australia and in the dry and wet zones of Sri Lanka^3–8^. At present, according to the database of the plant list (www.theplantlist.org, 2020), there are 30 plant name records matching with the query *“Paramignya*” in various geographical locations and native habitats.

In nature, the significant variation of *Paramignya* species in different geographical localities is one of the reasons leading to the uncertain taxonomic status^2,8–12^. Moreover, the taxonomy and the phylogeny of the genus *Paramignya* and other related genera of the subfamily Aurantioideae are complex due to their wide sexual compatibility via outcrossing, adventive type of apomixis or high frequency of somatic bud mutation^2,7,9,13,14^. These reasons lead to a blending of the phenotypical characteristics and the taxonomic misunderstanding or even taxonomic havoc in the genera of the subfamily Aurantioideae^7,15,16^. In addition, the taxonomic system for the classification of the genus *Paramignya* has been changed from time to time because the identification of the species depends predominantly on the morphological and geographical data^11,17^. Recently, DNA barcoding is an emerging technique for identification of many species based on short DNA regions specific for species. To date, various land plants including medicinal plants have been successfully identified by DNA barcodes^16–19^. The common DNA regions often selected as DNA barcodes for the investigation of taxonomy and the identification of many different species land plants were internal transcribed spacer (ITS), and three chloroplast regions maturase K (matK), ribulose-1, 5-bisphosphate carboxylase oxygenase large subunit (rbcL), and the intergenic spacer region trnH–psbA^16–19^. Recent case studies on the identification of medicinal plants based on DNA barcoding revealed that among universal barcodes, matK and ITS regions showed a high success rate of PCR amplification and discriminatory power followed by rbcL region. The trnH–psbA region provided low discriminatory power due to its low success rate of DNA sequencing^18,19^. Due to the complexity and uncertain morphological taxonomy of *Paramignya* species, the analysis of the molecular data, especially the DNA barcode sequences, are necessary for the identification and distinguish of these species. However, studies on the genetic variation and the phylogeny of the genus *Paramignya* using molecular data are relatively limited^11,16^.

Until now, reports on the genus *Paramignya* have predominantly focused on the investigation and the characterization of the physicochemical and the biopharmaceutical properties of the extracts^4,20–23^. A broad range of the value secondary compounds such as coumarin, tirucallane, acridone alkaloids, phenols, flavonoids, limonoid, sterols and derived glycosides was characterized as valuable resources for natural novel drug developments^4,20–30^. In recent years, the physicochemical properties, antioxidant, anti-proliferative and anti-inflammatory capacities of the leaf, stem, and root extracts of *P. trimera* were reported^24–27^. An abundant source of the natural compounds such as phenols, saponins, flavonoids, proanthocyanidins, and antioxidant agents was found in *P. trimera*^20,28,29^. The in vitro anticancer activity of the extracts of *P. trimera* against human pancreas and breast cancer cell lines MCF-7 via apoptosis induction was also investigated^25,30^. Although the phytochemical and biopharmaceutical properties of *P. trimera* were characterized, the morphology and the phylogenetic relationship of *P. trimera* with relatives were clearly undescribed. The high genetic diversity of the natural populations of *P. trimera* and the existence of the numerous local names make it difficult to identify and distinguish *P. trimera* from its relatives. Therefore, a well systematic analysis to identify and distinguish *P. trimera* from relatives in the family Rutaceae is necessary for the protection and the conservation of this plant.

In the present study, the morphological and DNA sequence data of the accessions of *P. trimera* and relatives distributing in Khanh Hoa and Lam Dong provinces were used to clarify the phylogenetic relation among these species. In particular, the morphological similarity between accessions of *P. trimera* and *A. buxifolia* was investigated to support for the hypothesis about the intergeneric hybrids of P. trimera with relatives. Additionally, the intraspecific and interspecific distances between accessions were also analyzed based on ITS, matK and rbcL sequences to discover a candidate DNA barcode for the identification and the discrimination of *P. trimera* from other species in the genus and in the related genera.

## Results

### Collecting plant specimens

**Figure 1:**
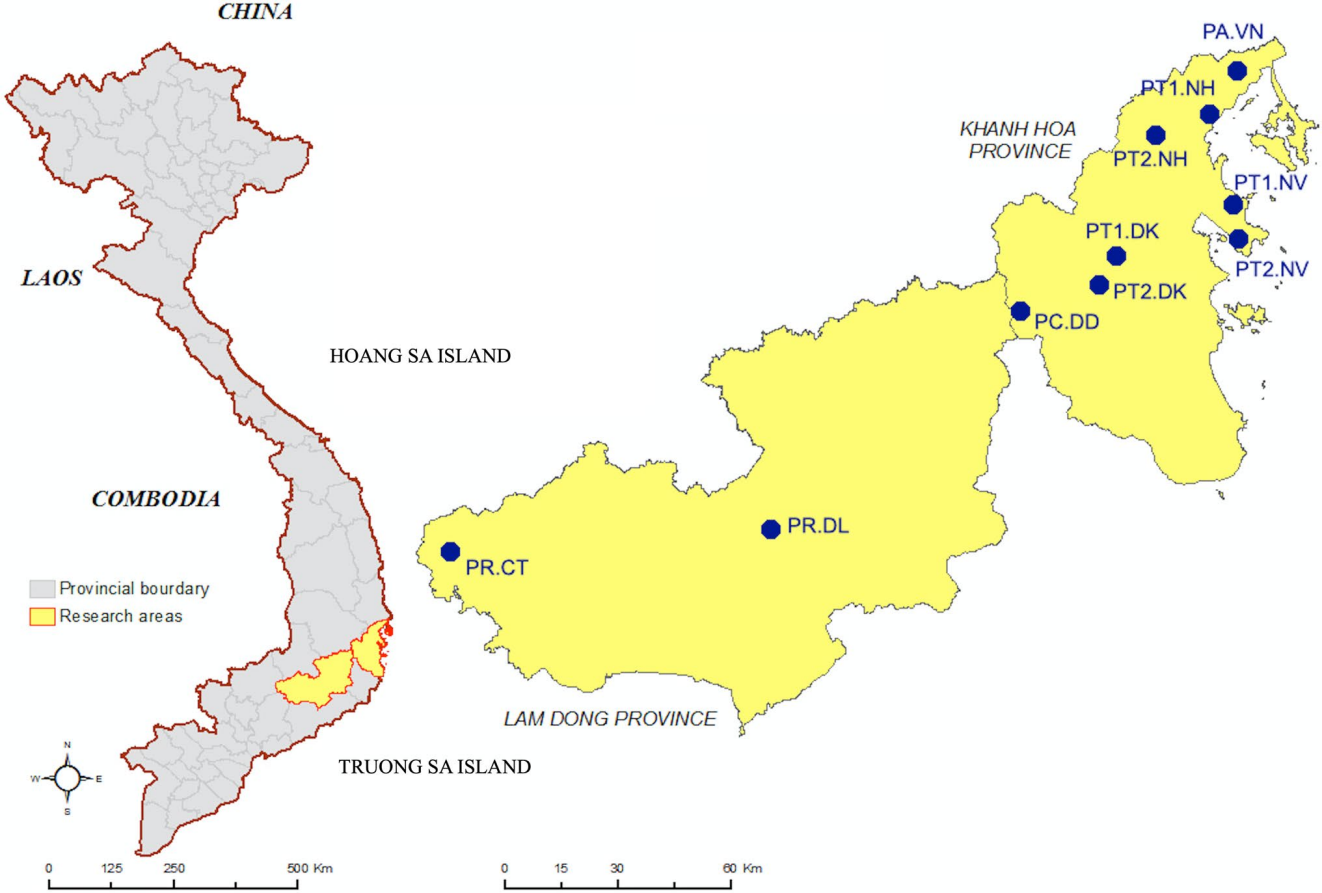
Map of the sampling sites. Accessions of species *P. trimera* (Oliv.) Burkill, *A. buxifolia* (Poir.) Oliv. ex Benth, *S. monophylla* (Lour.) Tanaka, and *L. scandens* (Roxb.), Wight were collected at sites displayed as circles in the map. The map was created by using ArcGIS 10.3 using the color rendering and grouping tools built-in and Paintbrush version 2.5 (20190914) on mac OS Catalina.

In the present study, 10 accessions assigned to 4 genera *Atalantia, Luvunga, Paramignya*, and *Severinia* were collected from different sites in Khanh Hoa and Lam Dong provinces of Vietnam (Fig. 1). Of these, six accessions of *P. trimera* (Oliv.) Burkill were collected at different sites in Khanh Hoa provinces including Ninh Van (PT1.NV, PT2.NV), Ninh Hoa (PT1.NH, PT2.NH), Dien Khanh (PT1.DK, PT2.DK); 1 accession of *A. buxifolia* (Poir.) Oliv. ex Benth collected in Van Ninh (PA.VN); 1 accession of *S. monophylla* (Lour.) Tanaka collected in Don Duong, Lam Dong province (PC.DD); two accessions of L. scandens (Roxb.), Wight, collected in Di Linh (PR.DL) and Cat Tien (PR.CT) in Lam Dong province (Fig. 1). The list of the collected accessions and information was summarized in Table 1.

**Table 1:** List of the collected accessions and information.

Sample sign	Geographic localities	Accession numbers of DNA sequences at NCBI			Longitude/Latitude
		ITS	matK	rbcL	
***Atalantia buxifolia (Poir.) Oliv. ex Benth(***^**1**^**)**					
PA.VN	Van Ninh, Khanh Hoa	MT193825	MT215526	MT215536	12°52′42″N, 109°22′52″E
***Luvunga scandens (Roxb.), Wight(***^**2**^**)**					
PR.DL	Di Linh Lam Dong	MT193832	MT215519	MT215530	11°40′18″N, 108°06′58″E
PR.CT	Cat Tien, Lam Dong	MT193830	MT215518	MT215535	11°46′18″N*,* 107°36′71″E
***Paramignya trimera (Oliv.) Burkill(***^**3**^**)**					
PT1.NV PT2.NV	Ninh Van, Khanh Hoa	MT193826 MT193827	MT215522 MT215524	MT215529 MT215528	12°38′80″N, 109°27′72″E
PT1.NH PT2.NH	Ninh Hoa, Khanh Hoa	MT193831 MT193833	MT215523 MT215525	MT215533 MT215532	12°36′65″N, 109°13′46″E
PT1.DK	Dien Khanh, Khanh Hoa	MT193829	MT215520	MT215531	12°06′10″N, 109°09′58″E
PT2.DK	Dien Khanh, Khanh Hoa	MT193834	MT215521	MT215534	12°20′22″N*,* 109°20′36″E
***Severinia monophylla (Lour.) Tanaka(***^**4**^**)**					
PC.DD	Don Duong, Lam Dong	MT193828	MT215517	MT215527	11°50′18″N*,* 108°54′63″E

(^1^)In our investigation, *A. buxifolia* (Poir.) Oliv. ex Benth was early described as *P. armata* var*. andamanica* King was found in Van Ninh (Khanh Hoa province).

(^2^)*L. scandens* (Roxb.), Wight was early described as *P. rectispinosa* Craib with accepted name *A. rectispinosa* (Craib) Engl.)

(^3^)*P. trimera* (Oliv.) Burkill distributed in different areas in Khanh Hoa province with the local name “Xao tam phan”.

(^4^)*S. monophylla* (Lour.) Tanaka was early described as *P. citrifolia* Oliv. with the local names “cam duong” or “quyt gai” found mainly in Don Duong (Lam Dong province).

### Taxonomic treatment

**Figure 2:**
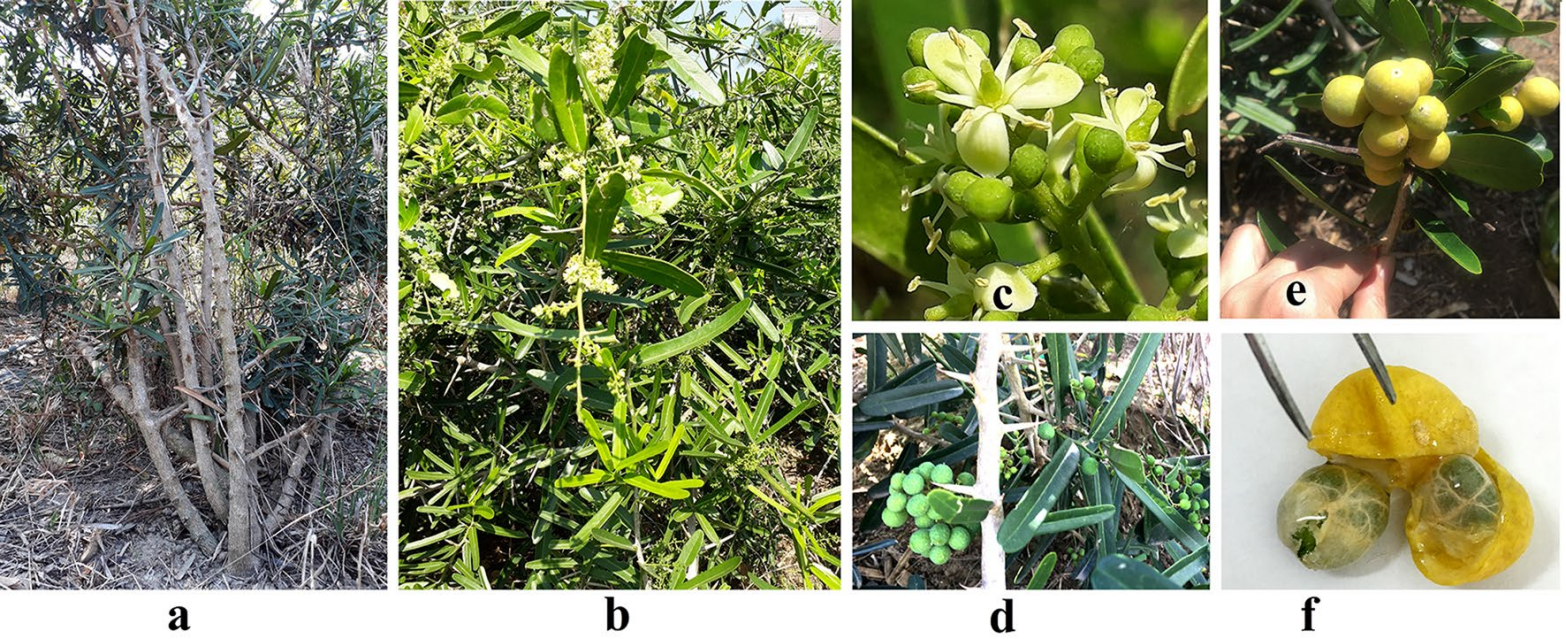
The typical morphology and anatomy of *Paramignya trimera* (Oliv.) Burkill. Woody shrub 1–4 m or above (**a**); A flowering tree (**b**); Typical trimerous flowers (**c**); Green fruits (**d**); Ripen fruits (**d**); Opened ripen fruit with two seeds encapsulated by mucus endocarp (**e**).

*P. trimera* (Oliv.) Burkill distributes in the high land areas in Khanh Hoa, Lam Dong provinces of Vietnam. *P. trimera* is scrambling shrub or erect, long, and curved spines, non-hairy stem. Leaves simple, typical narrow oblong, lamina 1.0–1.5 cm wide, 5–12 cm long; short petiole 0.5 cm long, leaf sub-vein 8–10 pairs; inflorescences axillary, fasciculate, peduncle 3–4 mm long, separate; calyx 3 lobes, 4 mm long; corolla 3; stamens 5, separate; ovaries 3, only 1 ovule, 2 locules in the ovary; globose fruit, 1.5–2.5 cm in diameter, 2 seeded. flowering time from May-Aug., fruiting Sep-Dec. Roots, leaves and stems were used as traditional medicine to treat liver diseases and cancers (Figs. 2, 4a).

*A. buxifolia* (Poir.) Oliv. ex Benth distributed mainly in Van Ninh (Khanh Hoa) with several local names such as “Xao cua ga” or “Quyt gai” are medium climbing shrubs, up to 3 m tall; branches grayish brown, branchlets green; *spikes axillary* 0.5–1.2 cm or sometimes unarmed, apex yellowish; leaves simple, 2.5–3.5 cm wide, 3.5–4.5 mm long, petiole 4–8 mm, leaf blade ovate, obovate, elliptic, glabrous, coriaceous, midvein slightly ridged, apex rounded to obtuse at tip; inflorescences axillary, 1 to several flowers. Flowers 5 merous, petals white, 3–4 mm, stamens 10, calyx persistent. Fruit bluish black when ripe, globose, slightly oblate, or subellipsoid, 7–10 mm in diam., smooth, 1 or 2 seeded. Flowering from May-Aug., fruiting Sep-Dec. Roots, leaves and stems were used as traditional medicine to treat cough, lung diseases and kidney disorders (Fig. 4b).

*S. monophylla* (Lour.) Tanaka found in Don Duong (Lam Dong) was thorny shrub or small tree; spikes axillary 1–1.5 cm; leaves simple, ovate, apex round or retuse at tip, coriaceous, glabrous, round at base, short petiole; Inflorescences 4–6-flowered; calyx ca. 3.5–5 mm long; petals 4, petals white, oblong, obtuse, glabrous, stamens 8–10; filaments ca. 12 mm long, glabrous; anthers ca. 5 mm long, linear; ovary ca. 2.5 × 1.5 mm, long-ovoid, glabrous, 3-locular; style ca. 7 mm long, continuous with ovary, cylindric, glandular, glabrous; stigma capitate ca. 2.5 mm broad, glandular. Fruits yellow to orange, globose 1.5–2.0 cm in diameter, 1–2 seeded; flowering time from May-Aug., fruiting Sep-Dec. This species was used effectively for cough, expectorant, fever, anti-inflammatory, sciatica treatment and prevent aging of skin cells, roots and leaves used for skin disease, burning leaves to kill mosquitoes and insects (Fig. 2c).

*L. scandens* (Roxb.), Wight was discovered in Lam Dong of Vietnam with the local name “Xao leo”. *L. scandens* is woody climber or scrambling shrub; rough tufted from the ground with strong axillary sharp straight or slightly recurved spines. Leaves compound, digitately trifoliate or bifoliolate or simple; petioles 2–6 cm long, glabrous; lamina ca. 6.0–18.0 × 2.5–4.0 cm, variable, oblong-elliptic or oblanceolate, cuneate at base, shortly acuminate at apex, coriaceous, glabrous; secondary nerves 15 pairs; branches brown puberulent. No information from flowering time has been described. According to traditional experience, this plant is used to treat rheumatism, liver disease and ascites (Fig. 2d).

### Phylogenetic relation analysis

The phylogenetic tree from ITS sequences included 3 groups (Fig. 5a). The first monophyletic group was only *S. monophylla* (PC.DD) as an out group. The second monophyletic group included 2 accessions of *L. scandens* (PR.DL and PR.CT). The third group was paraphyletic group with 9 accessions clustered in 2 sub-groups. The first sub-group included only *P. trimera*, whereas the second sub-group included 3 accessions *P. trimera* nested with *P. confertifolia* and *A. buxifolia*. In addition, in the second sub-group, the accessions of *P. trimera* collected in Dien Khanh, Vietnam (PT1.DK) and *P. confertifolia* from Mensong, China were in the same monophyletic clade whereas *A. buxifolia* (PA.VN) was clearly separated from others.

The unrooted tree from matK sequences included 3 groups in which the first monophyletic group were 2 species *P. lobata* and *P. scandens* (Australia), the second monophyletic group included only *P. confertifolia* (China) and the third group (paraphyletic group) included 3 sub-groups (Fig. 5b). The first sub-group included all accessions of *P. trimera*, the second sub-group included only *S. monophylla* and the third sub-group included *L. scandens* and *A. buxifolia*.

The unrooted tree from rbcL sequences included 2 main groups in which the first group included 3 species *P. scandens, P. monophylla* and *P. lobata* (Australia) and the second group (paraphilic group) included 5 species *P. trimera, P. confertifolia* (China), *S. monophylla* (Japan), *A. buxifolia*, and *L. scandens* (Fig. 5c). In this group, some accessions of *P. trimera* were nested in the paraphylic sub-groups because they did not share an immediate common ancestor.

The pattern of the phylogenetic tree constructed from the concatenated sequences was similar to that of ITS sequences (Fig. 5d). The tree included one monophyletic group with only *L. scandens* and one paraphyletic group with the accessions of *P. trimera* nested within *P. confertifolia*, *A. buxifolia* and *S. monophylla*.

### Genetic distance analysis

The overall genetic distances for ITS, matK, rbcL and concatenated sequences were 0.11 ± 0.01, 0.29 ± 0.02, rbcL 0.48 ± 0.05 and 0.05 ± 0.0, respectively (Table 2). An overlap between the maximum intraspecific distances and the minimum interspecific distances were observed in the cases of ITS, rbcL and concatenated sequences (Table 2, Fig. 6a,c,d). In case of matK, a clear barcode gap was found between the maximum intraspecific distance (0.0028) and the minimum interspecific distance (0.0056). The histogram and ranked pairwise (K2P) distances demonstrated a significant difference in the cases of matK and rbcL (Fig. 6b,c).

**Table 2:** Intraspecific and interspecific distances across all data.

	Genetic distances							
	ITS		matK		rbcL		Concatenated sequence	
	Intra. specific	Inter. specific	Intra. specific	Inter. specific	Intra. specific	Inter. specific	Intra. specific	Inter. specific
Overall mean distance	0.11 ± 0.01		0.29 ± 0.02		0.48 ± 0.05		0.05 ± 0.0	
Minimum	0.0061	0.0184	0.0000	0.0056	0.0125	0.0062	0.0056	0.0293
Maximum	0.2205	0.2131	0.0028	1.309	0.0712	1.3302	0.0843	0.0474
Average	0.1122 ± 0.0586	0.0985 ± 0.0547	0.0014 ± 0.0012	0.6806 ± 0.4820	0.45 ± 0.05	0.7521 ± 0.5933	0.0468 ± 0.0223	0.03147 ± 0.0056

## Discussion

### The controversy of the morphological classification

According to the database of the plant list (www.theplantlist.org, 2020), 30 plant name records were matched with the query *Paramignya*. In the BOLD system, 10 published specimen records represented for 4 *Paramignya* species (*P.* cf. *scandens*, *P. mindanaensis*, *P. lobata* and *P. confertifolia*) and 6 barcodes sequences were described and linked to NCBI database.

**Figure 3:**
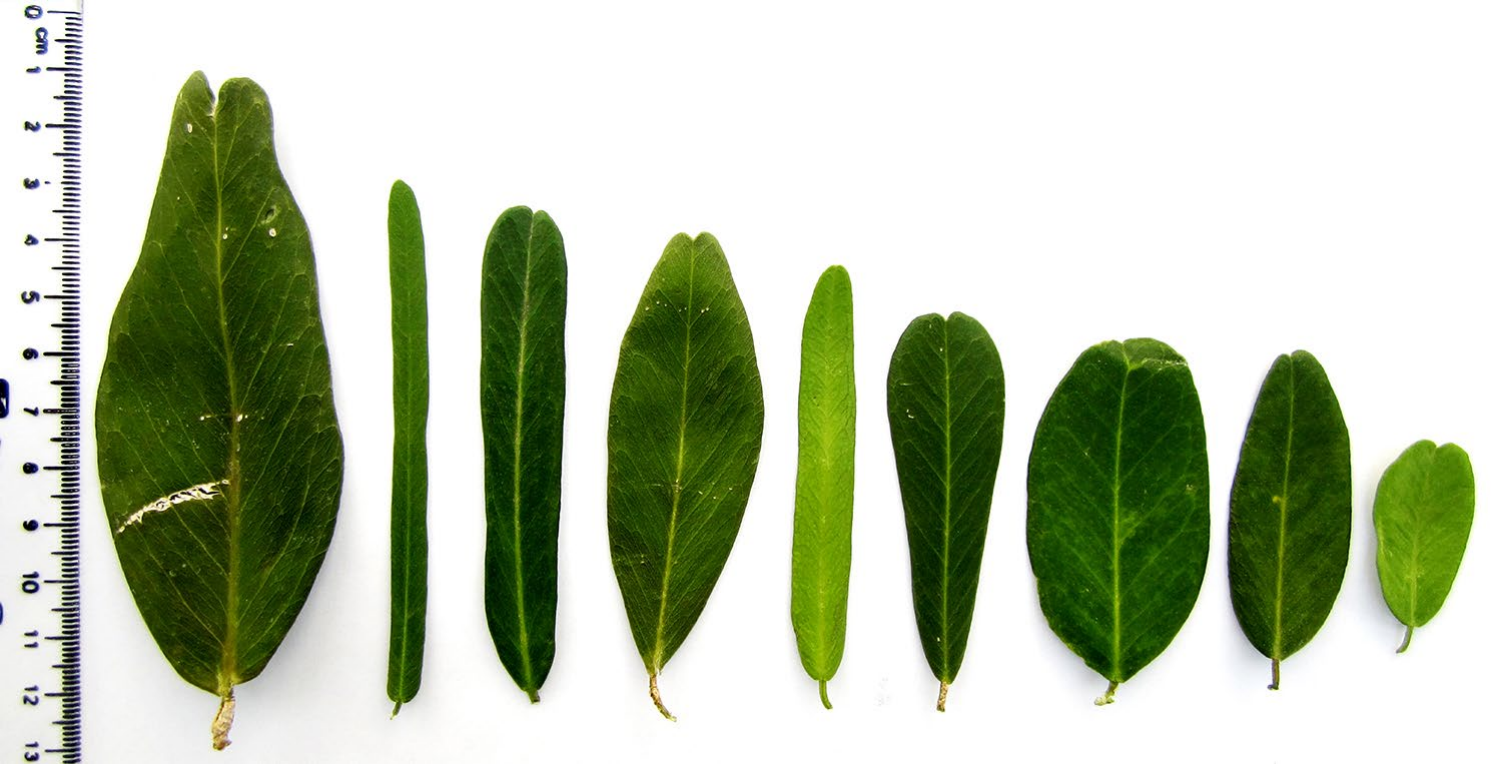
The morphological variation of leaves among accessions of *P. trimera* (Oliv.) Burkill. Leaves of *P. trimera* showing oblong, elongate, obovate, ovate, oval, elip, roundish forms with rounded or obtuse apexes. Different forms of leaves were sometimes found in a single individual plant.

**Figure 4:**
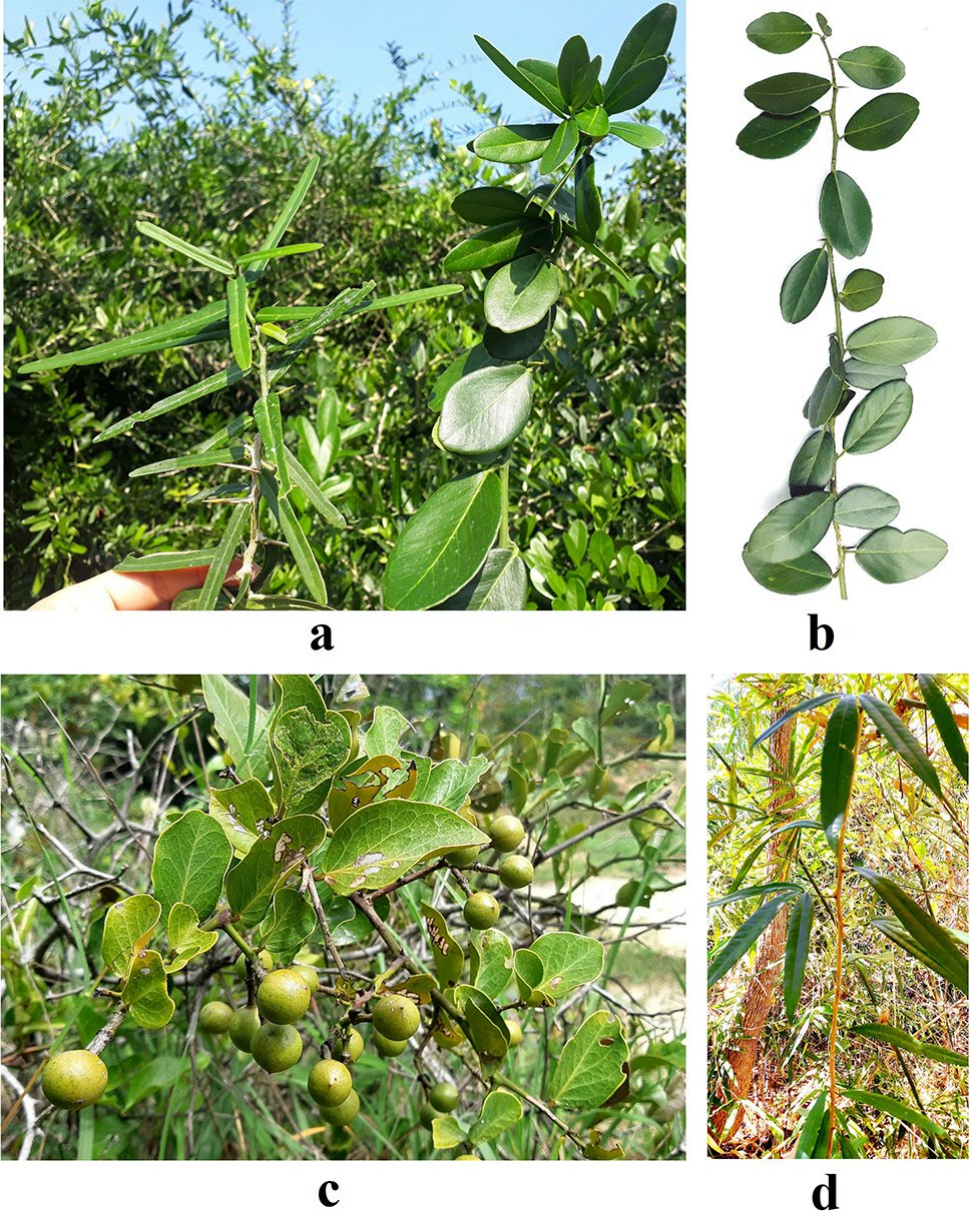
Morphological characteristics of four species of different genera. A single individual plant of *P. trimera* with two different forms of leaves (**a**), typical morphology of *A. buxifolia* (**b**), the scrambling shrub of *S. monophyla* with fruits (**c**), and the typical morphology of *L. scandens* with different types of leaves (**d**).

**Figure 5:**
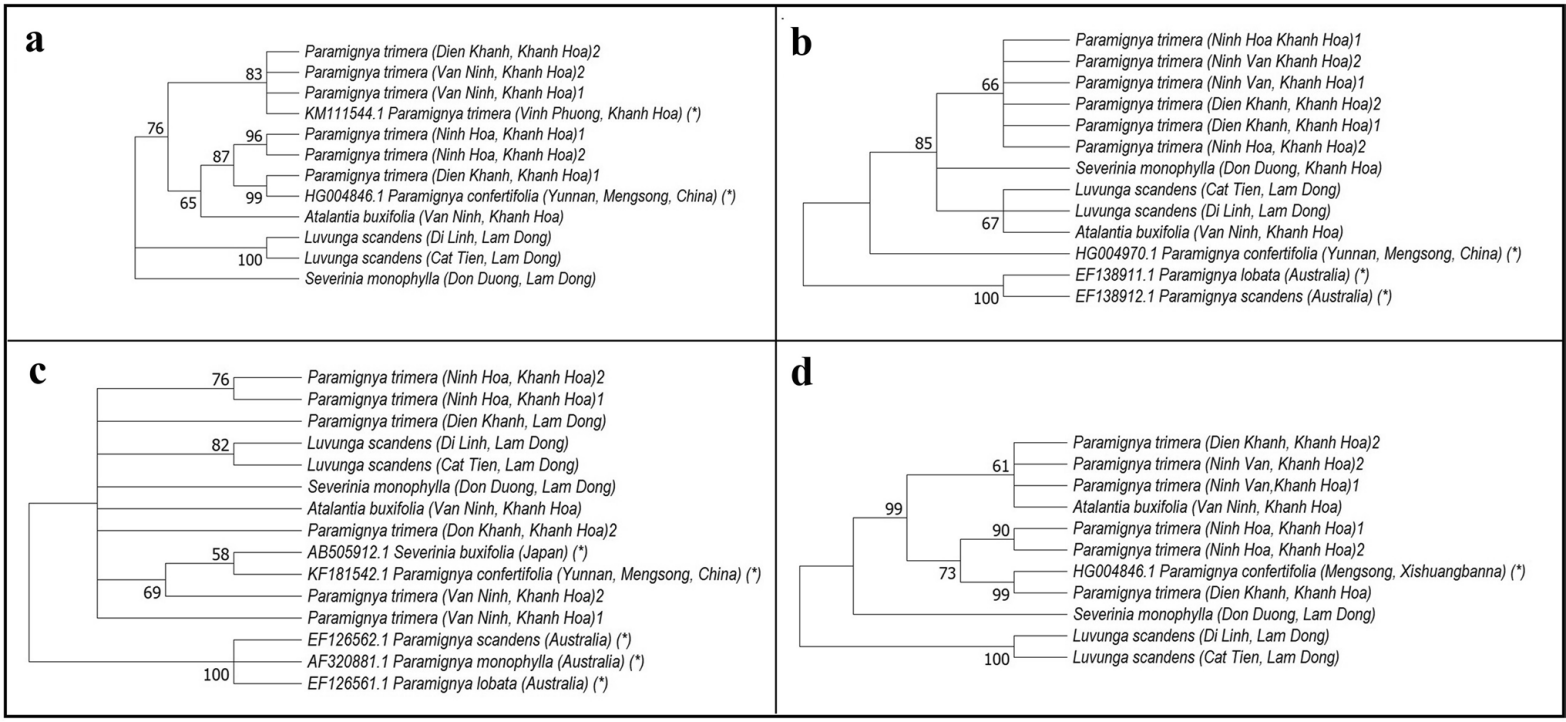
Condensed cladogram showing phylogenetic relationships of the accessions of *P. trimera* and relatives from DNA barcode sequences. Trees constructed from ITS (**a**), matK (**b**), rbcL (**c**) and concatenated sequences (**d**) using maximum likelihood (numbers at nodes are maximum likelihood bootstrap support values; branches with bootstrap support < 60 collapsed). The sequence data retrieved from GenBank with accession numbers were marked with asterisks (*).

This study is the first to sample all accessions of *P. trimera* (Oliv.) distributing in Khanh Hoa and the relatives in Khanh Hoa and Lam Dong provinces of Vietnam. By morphology analysis, accessions *P. trimera* were different from other *Paramignya* species because of its trimerous flowers with 3 corollas (Fig. 2c)^1,31^. However, a wide range variation in the characteristics of the leaves of *P. trimera* was observed (Fig. 3). In addition, some accessions of *P. trimera* with their leaves similar to *A. buxifolia* that caused uncertainty about the taxonomic classification (Fig. 4a,b). The blending forms of leaves found in some accessions of *P. trimera* collected in Khanh Hoa province suggested that they were likely the “graft chimera” or the intergeneric hybrids between *P. trimera* and the closely relatives such as *A. buxifolia* or *S. monophylla* (Fig. 4). The lack of information on the holotype or lectotype of *P. trimera* in combination with the complexity of the nexus between self-incompatibility, apomixis and outcross led to the difficulty in the discrimination of the hybrid lines of *P. trimera* with its relatives. However, based on the phylogenetic relationships demonstrated in the cladograms (Fig. 5), the “graft chimera” or hybrid lines would be the results of the outcrossing between *P. trimera* and *A. buxifolia* rather than *S. monophylla* because *P. trimera* and *A. buxifolia* were clustered in one clade.

For years, in term of taxonomy, most *Paramignya* species were listed in the genus *Atalantia*^30^. *P. trimera* used to have other synonyms such as *Triphasia monophylla* DC or *A. recurva* Benth^3,6^ or possibly congeneric with *Luvunga* species^7^. According to Mabberley, *Paramignya* species were so closely related to the genus *Luvunga* that they were listed in the genus *Luvunga* with the scientific name *L. monophylla* (DC.)^7^. However, in this study, accessions of *P. trimera* were separated from *Luvunga* in all DNA barcode sequences. In the aspect of morphology, the genus *Luvunga* Wight & Arn was held to differ from *Paramignya* in its 3–5 corollas, 6–10 stamens and 2–4 locules in the ovary^6^. Since 1931, Burkill has also named *A. trimera* as *P. trimera* because *Paramignya* species have small fruits containing fluid mucus and without pulp vesicles ^3^. However, the misidentification of *Paramignya* species was sometimes due to the characteristics of their leaves or axillary spines^31^. The simple leaves of some species of the genus *Paramignya*, including *P. trimera,* sometimes also occur in the genus *Luvunga* because of their petioles shorter than those of the usual trifoliate leaves (Fig. 4d). This remark was also mentioned in the notes on the genus *Paramignya*^6^*.* According to the study on the phylogenetic relationships of the sub-family Aurantioideae inferred from chloroplast DNA sequence data, in the tribe Citreae, nearly all the species develop axillary spines, single or paired, sometimes curved as in *Luvunga* and *Paramignya*^31,32^. In our study, some accessions of *P. trimera* were similar to *A. buxifolia,* especially their leaves and stems (Fig. 4a,b). This was the reason for the difficulty in providing an unequivocal identification for *Paramignya* species. However, based on both morphological and DNA sequence data, it was possible to distinguish *P. trimera* from other species of the genus *Paramignya*.

### Analysis of DNA barcode

At the time of this study, in the NCBI Entrez system, there are 39 records matched with the query “Paramignya” including *P. lobata, P. scandens, P. monophylla, P. trimera and P. confertifolia*. A total of 26 barcode sequences of ITS1, ITS2, matK, ribulose-1,5-bisphosphate carboxylase/oxygenase gene large unit (rbcL), hyper-variable regions of chloroplast ribosomal protein S (Rps4, Rps16), psbA-trnH, ATPase beta-subunit gene (atpB), trnG, trnF and trnD were found in the GenBank. For the phylogenetic tree from ITS sequences, most accessions assigned to *P. trimera* were clustered with *P. trimera* (KM111544.1), the other accessions of *P. trimera* were nested within a clade with *P. confertifolia* (HG004846.1) (Fig. 5a). This problem was likely due to the paraphyly or polyphyly of the conspecific DNA sequences that caused by incomplete lineage sorting or the existence of the interspecific hybrids among these closely related species. This result also matched with the overlap of the distance between the maximum intraspecific and the minimum interspecific distances (Table 2). Although the ITS data were not completely support for the identification and the discrimination of all species of the genus *Paramignya*, the tree created by ITS sequences supported for the classification of *P. trimera* and relatives. These results were also consistent with the notes in the sub-family Aurantioideae (Rutaceae)^6^.

The tree created by matK sequences supported Swingle and Reece’s classification of subfamily^1^. The results were matched with the notes about molecular phylogeny of the orange subfamily using cpDNA sequences^2^ and genetic relationships of citrus and its relatives^16^. The clear barcode gap between the maximum intraspecific distance and the minimum interspecific distance (Table 2) also agreed with the phylogenetic tree. It suggested that matK was a candidate DNA barcode for the classification and the discrimination of *P. trimera* from other relatives in the genus *Paramignya* and other genera in the sub-family Aurantioideae. The presence of the paraphyletic groups in the phylogenetic tree based on rbcL sequences partly reflected the unresolved relationships of closely related taxa. These results were also matched with the data of intraspecific and interspecific distances (Table 2). The overlap between the intraspecific and interspecific distances among *P. trimera* with *S. buxifolia* and *S. monophylla* suggested that rbcL would not be suitable barcode marker for the classification and the discrimination of *P. trimera* from other *Paramignya* species as well as other genera. The results were also matched with those in the study on the molecular phylogeny of the subfamily Aurantioideae using cpDNA sequences^2^. The overlap between the maximum intraspecific distance and the minimum interspecific distance suggested that the accessions of *P. trimera* with intermediate forms of leaves were likely the interspecific hybrids among *P. trimera* with *A. buxifolia* (Fig. 4c,d).

**Figure 6:**
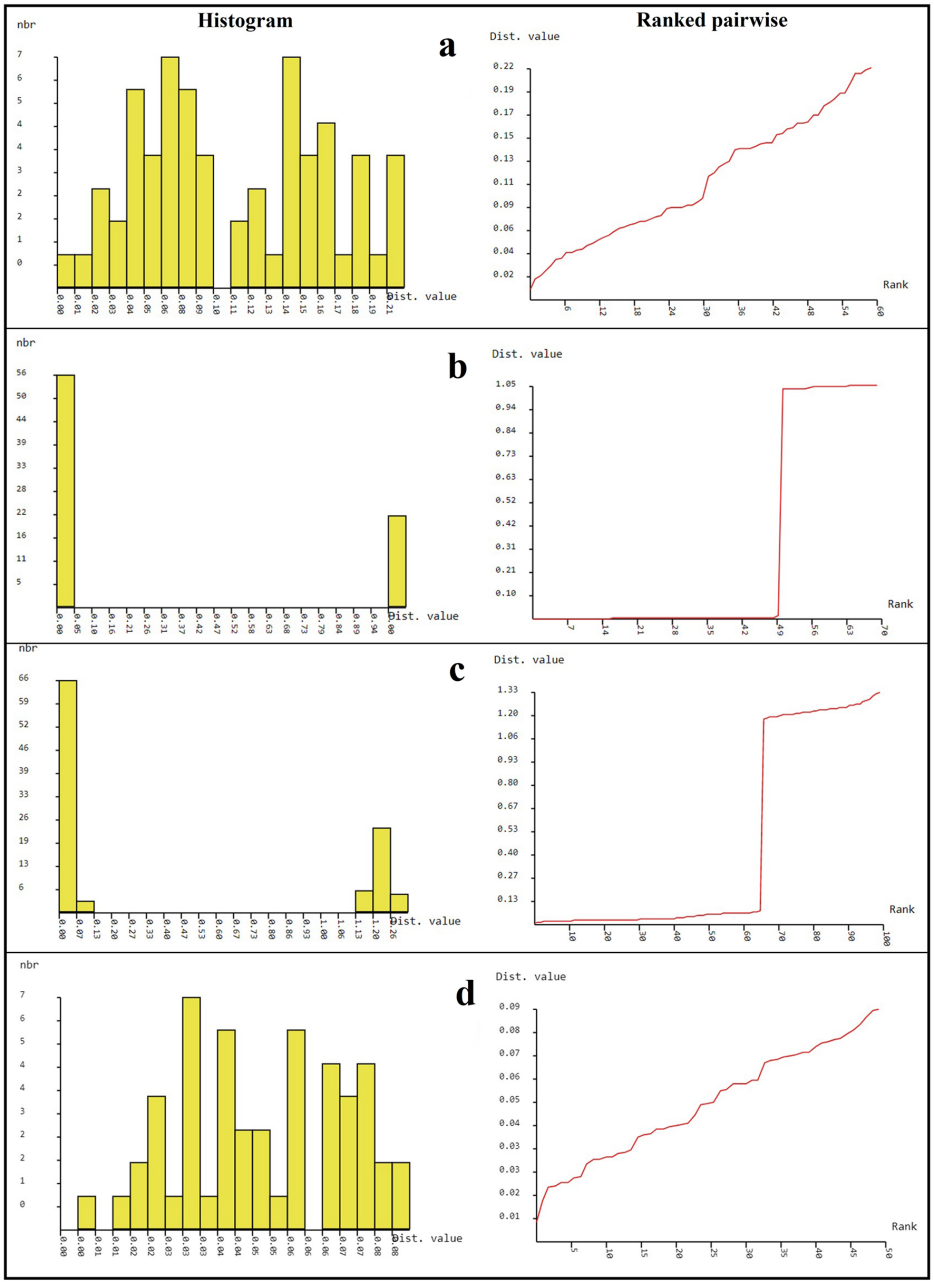
Histogram and ranked pairwise (K2P) distances among barcode sequences by automated barcode gap discovery (ABGD) approach. The analysis of barcode gap using ITS (**a**), matK (**b**), rbcL (**c**) and concatenated sequences (**d**) of *P. trimera* and relatives.

Based on both the morphological and DNA sequence data of *P. trimera* and relatives, we suggested that the identification and the discrimination of *Paramignya* species by using DNA barcodes was reliable only if a significant difference was consistently detected between the maximum intraspecific distance and the minimum interspecific distance. The use of the mean instead of the minimum interspecific distance could exaggerate the size of the “barcoding gap” and lead to misidentification^33^. Therefore, in the case of *P. trimera* the approach to reliably detect the barcoding gap is to determine the gap between the maximum intraspecific and the minimum interspecific distances^33^. Therefore, only matK sequence would be a suitable candidate DNA barcode for the identification and the discrimination of *P. trimera* from other *Paramignya* species (Table 2). Although the histogram and ranked pairwise (K2P) distances analyzed by ABGD program showed a clear gap in both cases of matK and rbcL (Fig. 6b,c), the barcode gap was only found in the case of matK rather than rbcL. Other DNA sequences including ITS, rbcL and concatenated sequences proved inefficient to solve the relationships within the *Paramignya* species and some close relatives such as *P. confertifolia*, *L. scandens*, *A. buxifolia* and *S. monophylla* (Table 2 and Fig. 5). In comparison to matK and rbcL sequences, although ITS showed higher mutation rate and more informative sites (data not shown), it was likely not suitable for sufficiently developing a DNA barcode to distinguish between *Paramignya* species. Although this comparison was relative because of the available heterogeneous datasets, the results provided additional insights into the effective of DNA sequences as barcode markers for accurate identification of *P. trimera* as well as *Paramignya* species. This consistent problem was due to some factors such as the inadequate number of samples in different geographical locations, the shortage of both morphological and molecular data of well-characterized phylogeny, or the interspecific hybrids as a result of the outcrossing enforced by self-incompatibility. It was likely that the wide sexual compatibility of the genus *Paramignya* and other genera of family Rutaceae was also one of the reasons leading to the difficulty in taxonomic identification. Although the comprehensive database such as BOLD system has been grown up rapidly, few well-sampled datasets, especially for the genus *Paramignya* are available to test its efficient performance. Thus, the considerable promise of barcoding will be realized only if the solid taxonomic foundations were well understood and established thoroughly sampled clades. Obviously, DNA barcoding is a system for species identification by using a short-standardized sequence as a “barcode” to assign an unknown specimen to a known species, however, a question on which DNA region can be used as the standard barcode should be adequately addressed for *P. trimera* as well as other species of the genus *Paramignya*.

Based on the obtained results we suggested the use of DNA barcodes was helpful to identify and distinguish of *Paramignya* species. In addition, the combination with other data there would allow to minimize the probability of misidentification. Therefore, the further systematic study and species identification of *Paramignya* are still needed to provide reference data for the screening of DNA barcode and the species discrimination that could provide theory basis for the identification and conservation of valuable medicinal plants.

## Conclusion

It was the first time the morphology and the phylogenetic relation of *P. trimera* and some relatives of the family Rutaceae collected from Khanh Hoa and Lam Dong provinces of Vietnam were analyzed. A combination of morphological data, BOLD platforms and DNA barcode sequences was efficiently support for the identification and the discrimination of *P. trimera* from its relatives. In addition, the presence of the intermediate forms of *P. trimera* was likely the interspecific hybrid lines as the results of the outcross between *P. trimera* with closely related species, notably *A. buxifolia.* It also suggested that the wide sexual compatibility could lead to the difficulty in taxonomic identification of *P. trimera* and *Paramignya* species. The study supported for the accurate identification for exploitation and of *P. trimera* in Vietnam as a valuable indigenous source of medicinal plant.

## Materials and methods

### Plant materials

A total of 10 accessions representing naturally distributive populations of *P. trimera* and related species in the regions of Ninh Van, Ninh Hoa, and Dien Khanh (Khanh Hoa province) and Di Linh, Cat Tien, Don Duong (Lam Dong province) was sampled across their original habitats between Nov. 2017 to Oct. 2019 (Table 2). The geographical locations of the sites were noted in Table 2. The chosen accessions for sampling were separated from each other at a distance of at least 5 km. The collected accessions were identified based on the traditional taxonomic keys, specimen collection stock photos and images available at GBIF (the Global Biodiversity Information Facility), databases of the Plant list (a working list of all plant species), botanical descriptions^1^ and the book “An Illustrated Flora of Vietnam”^34^. Fresh leaves were photographed at collection sites and stored in sealed bags with silica-gel at normal condition within 2 days until DNA extraction.

### DNA extraction

Total genomic DNA was extracted from 1.0 g leaf material following CTAB method^35^ and then purified by using the Thermo Scientific GeneJET Genomic DNA Purification Kit (# K0722). DNA quality was examined by electrophoresis in 1% agarose and quantified with a Nanodrop (Eppendorf, USA). The purified genomic DNA was stored at − 20 °C for PCR amplification.

### Amplification of barcode regions

ITS region was performed by using universal primers for plant (ITS-p5: CCTTATCAYTTAGAGGAAGGAG and ITS-p4: CCGCTTAKTGATATGCTTAAA^19^. The 25 μl reaction mixture contained 100 μM NTPs, 0.1 μM each primer, 1X PCR buffer with 1.5 mM MgCl_2_, 2 μl of template DNA sample, and 1 U of Taq DNA Polymerase (Qiagen, Cat. No. 201203). For amplification of ITS sequences, DNA templates sometimes were diluted to a final concentration of 10 ng µL^−1^ and the annealing temperature was optimized with a gradient PCR to obtain specific band. The thermal cycler was programmed to perform an initial cycle of denaturation at 94 °C for 4 min, followed by 30 cycles of 30 s at 94 °C, 30 s at 50–55 °C, 90 s at 72 °C. A final step was done by a 10 min extension at 72 °C to allow completion of unfinished DNA strands.

Similar PCR conditions were applied for the amplification of matK and rbcL sequences. The matK sequences of all accessions were amplified by using the universal primers matK-390F TAATTTACRATCAATTCATTCAATATTTCC and matK-1326R GARGAYCCRCTRTRATAATGAGAAAGATTT according to Kyndt, T. et al. 2005 with 52 °C annealing temperature^36^. The rbcL sequences of all accessions were amplified by using the universal primers rbcL-F ATGTCACCACAAACAGAGACTAA and rbcL-R TTCGGCACAAAATACGAAACGATCTCTC with 56 °C annealing temperature^37^.

PCR products were examined by electrophoresis and purified by using the QIAquick PCR Purification Kit (Qiagen, Cat. No. 28104) following the manufacturer’s protocols. Purified PCR products were directly sequenced in both directions using the ABI PRISM dye terminator cycle sequencing ready kit with AmpliTaq DNA Polymerase (Applied Biosystems Inc.). Unincorporated dye terminators were removed using the DyeEX Dye-Terminator removal Kit (Qiagen, Cat. No. 63204) following the manufacturer’s recommendations. Sequencing samples were automatically loaded and injected on the ABI 3500 XL (Applied Biosystem Inc.) following the instruction of the manufacture.

### Sequence splicing and correction

Both forward and reverse nucleotide sequences were visualized and aligned using BioEdit (Ver. 7.0.5.3). Sequences were checked manually to find sequencing errors, if any, to correct. Erroneous and ambiguous base calls with low quality were trimmed from both ends. BLAST searches were performed for consensus sequences to identify best matches in GenBank at NCBI. In this study, the sequences of ITS, matK and rbcL have been deposited in GenBank as phylogenetic data under the accession numbers MT215517-MT215536 and MT193825- MT193834 (Table 1). Based on multiple sequence alignment, the datasets of ITS, matK and rbcL sequences of *Paramignya* species were pruned to a maximum of 715, 774 and 570 nt, respectively. The combined datasets as the concatenated sequences (ITS + matK + rbcL) of accessions were 2059 nt.

### Genetic distance and phylogenetic analysis

The nucleotide divergence was estimated based on the multiple sequence alignment of ITS, matK, rbcL and the concatenated sequences by MEGA X (Ver.10.1.7) using the Kimura-2-parameter (K2P) model^38^. A uniform distribution was set as rate variation among sites. The Maximum likelihood (ML) trees were generated for each DNA sequence separately and combined as concatenated sequences by using MEGA X software with 1000 bootstrap replications^39,40^. The tree with the highest log likelihood is shown and the percentage of trees in which the associated taxa clustered together is shown next to the branches. Due to the significant small value of the branch lengths that may affect the display of trees, all trees in this study were plotted as cladograms. The cut-off value for condensed trees were set at 50% to better represent hypothetical phylogenetic systematics relationship among accessions. Gaps and missing data treatment were selected as partial deletion with 95% site coverage cutoff (including alignment gaps, missing data, and ambiguous bases were allowed at any position).

The overall genetic distances estimated for the ITS, matK, rbcL and concatenated sequences were estimated by MEGA X software. To determine the barcoding gap between pairwise genetic distances among and within species, the intraspecific and interspecific distance were calculated by ExcaliBAR program based on the original distance matrices computed by MEGA-X software. The barcoding gap was calculated by the difference between the maximum intraspecific distance and the minimum interspecific distance^33,41^. The automatic barcode gap discovery (ABGD) (http://wwwabi.snv.jussieu.fr/public/abgd/abgdweb.html) was used to generate distance histograms and distance ranks with two X values of relative gap width (1.0 and 1.5) and distance metric (K2P)^42^. Default values were employed for all other parameters, P (prior intraspecific divergence) ranged from 0.001 to 0.1 while Steps was set to 10, and Nb bins (for distance distribution) was set to 20^42^.

### Map of the sampling sites

The map of sampling sites was created by ArcGIS 10.3 using the color rendering and grouping tools built-in. The collection sites and names were placed on the map based on actual coordinates by using Paintbrush version 2.5 (20190914) on mac OS Catalina.

### Informed consent for publication

The authors agree to publish the information and image(s) in an online open-access publication.

## Supplementary information

Supplementary Information.
