# Supplementary material for: Phylogenetic relationship of Paramignya trimera and its relatives: an evidence for the wide sexual compatibility
Source: Sci Rep. 2020 Dec 10;10:21662. doi: 10.1038/s41598-020-78448-2 (PMC7730421; doi:10.1038/s41598-020-78448-2)
Supplement: Supplementary file 1 — Supplementary Information. [file 41598_2020_78448_MOESM1_ESM.docx]

**PACKAGE DATA FOR ONLINE RESOURCES**

**Online Resource 1**

*Paramignya trimera (KM111544.1)*

**Paramignya trimera internal transcribed spacer 1, partial sequence; 5.8S ribosomal RNA gene and internal transcribed spacer 2, complete sequence; and 28S ribosomal RNA gene, partial sequence**

GenBank: KM111544.1

[GenBank](https://www.ncbi.nlm.nih.gov/nuccore/KM111544.1?report=genbank) [Graphics](https://www.ncbi.nlm.nih.gov/nuccore/KM111544.1?report=graph)

>KM111544.1 Paramignya trimera internal transcribed spacer 1, partial sequence; 5.8S ribosomal RNA gene and internal transcribed spacer 2, complete sequence; and 28S ribosomal RNA gene, partial sequence

TCGGAGTGTTGTTTTTTTGTATTTTACTGCCAGCAGACGACCCGCGAACCAGTAAGAGACCACTGGCGGC

GGGAGGGGGGGCGCGCTCCTCGCGGGCGCTCCTCCTTCCCCCCCAAAATCGCCTCTGGGAGGGGGACTCG

TCCCTCTCCCGGCCGGCGAAACAACGAACCCCCGGCGCGGACTGCGCCAAGGAAATCCAACGAGAGAGCA

CGCTCCCGCGGCCCCGGAGACGGGTTGCCGCGGGGTGCGCCGCCTTCTTTCACATGTATCCAAAACGACT

CTCGGCAACGGATATCTCGGCTCTCGCATCGATGAAGAACGTAGCGAAATGCGATACTTGGTGTGAATTG

CAGAATCCCGTGAACCATCGAGTCTTTGAACGCAAGTTGCGCCCAAAGCCGTTAGGCCGAGGGCACGTCT

GCCTGGGTGTCACGCATCGTTGCCCCACCCCACCCCCCCGGACCAAGGCGGGGGCCCGGAGGTGCGGGCG

GAGATTGGCCTCCCGTGCGCCGAACGCTCGCGGTTGGCCCAAATCCGAGTCCTCGGCGACCGAAGCCGCG

GCGATCGGTGGTGAAAGAAAAGCCTCTCGAGCTCCCGCCGCGCGCTCGGTCTCCGCGAGGGGACCCTGTG

ACCCTAACGCTCCGCGCAAGCGGAAGCCAGCATCGCGACCCCAGGTCAGGCGGGATCACCCGCTGAGTTT

AAGCATATCAATAAGCCGGAGGAA

*Paramignya confertifolia* (KF181542.1, HG004970.1, HG004846.1)

# Paramignya confertifolia isolate 372_2_17 ribulose-1,5-bisphosphate carboxylase/oxygenase large subunit (rbcL) gene, partial cds; chloroplast

GenBank: KF181542.1

[GenBank](https://www.ncbi.nlm.nih.gov/nuccore/KF181542.1?report=genbank) [Graphics](https://www.ncbi.nlm.nih.gov/nuccore/KF181542.1?report=graph) [PopSet](https://www.ncbi.nlm.nih.gov/popset?DbFrom=nuccore&Cmd=Link&LinkName=nuccore_popset&IdsFromResult=513130715)

>KF181542.1 Paramignya confertifolia isolate 372_2_17 ribulose-1,5-bisphosphate carboxylase/oxygenase large subunit (rbcL) gene, partial cds; chloroplast

ACAGAGACTAAAGCGAGTGTTGGATTCAAGGCCGGTGTTAAAGATTATAAATTGACTTATTATACTCCTG

ACTATGTAACCAAAGATACTGATATCTTGGCAGCATTCCGAGTAACTCCTCAACCCGGAGTTCCACCCGA

GGAAGCGGGGGCTGCGGTAGCTGCGGAATCCTCTACTGGTACCTGGACAACTGTGTGGACCGATGGGCTT

ACCAGCCTTGATCGTTACAAAGGGCGATGCTACAACATTGAGCCCGTTGCTGGAGAAGAGAATCAATATA

TATGTTATGTAGCTTACCCGTTAGACCTTTTTGAAGAAGGTTCTGTTACTAACATGTTTACTTCCATTGT

GGGTAATGTATTTGGTTTCAAAGCACTGCGCGCTCTACGTCTAGAGGATCTACGAATCCCTCCTGCGTAT

TCTAAAACTTTCCAAGGCCCGCCTCACGGCATCCAAGTTGAGAGAGATAAATTGAACAAGTATGGCCGTC

CCCTGTTGGGATGTACTATTAAACCTAAACTGGGGTTATCCGCTAAGAATTATGGTAGGGCGGTTTATGA

ATGTCTACGC

# Paramignya confertifolia chloroplast partial matK gene for maturase K, specimen voucher HITBC:Liana Mengsong 372_2_17

GenBank: HG004970.1

[GenBank](https://www.ncbi.nlm.nih.gov/nuccore/HG004970.1?report=genbank) [Graphics](https://www.ncbi.nlm.nih.gov/nuccore/HG004970.1?report=graph)

>HG004970.1 Paramignya confertifolia chloroplast partial matK gene for maturase K, specimen voucher HITBC:Liana Mengsong 372_2_17

CGCGAATGGGTAAAGGATGCCTCTTCTTTACATTTATTACGGTTCTTTCTCCACGAGTATTTTAATTCGA

ACAGTCTTATTACTCCAAAGAATTCTATTTCTGTTTTTTTAAAAAGTAATCCAAGATTTTTATTGTTTCT

ATATAATTCTCATGTATATGAATATGAATCCATCCTCTTTTTTCTCTGTAACCAATCGTCTCATTTACAA

TCAACATCCTCTCGAGTCCTCGTTGAGCGAACGTATTTCTATGGAAAAGTCGAACATCTTGTCGAAGTCT

TTGCTAAAGATTTTCAGGACATCTTAGGGTTCTTCAAGGATCCTTTCATGCATTATGTTAGATATCAAGG

AAAATCCATTTTGGCTTCAAAGGATACGCCTCTTCTGATGAATAAATGGAAATATTACCTTGTCGGTTTA

TGGCAATGGCATTTTCACGTGTCTTCTCAACCAGGAAGGGTTCAGCTAAACCACTTACACTTAGGCAAGT

ACGCTATTAACTTTCTGGGCTATCTTTCCGGTGTGCGACTAAATTCTTTGTTGGTACGGAGTCAAATGCT

AGAAAATTCATTTCTAATAGGTAATTCTATGAAGAAGGTCGATACGACCGTTCCAATTATTCATCTGATT

GGATCATTGACTAAGGCGCGGTTTTGTAACGCATTAGGGCATCCTATCAGTAAGTCGACTTGGGCCGATT

TCTCTGATTCTCATCTTATCGACCGATTTGTGCGTATATGCAGAAATCTTTCTCATTATTACAGCGGATC

TTCAAAAAAAAAAAGTTTGTATCGAGTAAAATATATACTTCGG

# Paramignya confertifolia genomic DNA containing 18S rRNA gene, ITS1, 5.8S rRNA gene, ITS2 and 28S rRNA gene, specimen voucher HITBC:Liana Mengsong 372_2_17

GenBank: HG004846.1

[GenBank](https://www.ncbi.nlm.nih.gov/nuccore/HG004846.1?report=genbank) [Graphics](https://www.ncbi.nlm.nih.gov/nuccore/HG004846.1?report=graph)

>HG004846.1 Paramignya confertifolia genomic DNA containing 18S rRNA gene, ITS1, 5.8S rRNA gene, ITS2 and 28S rRNA gene, specimen voucher HITBC:Liana Mengsong 372_2_17

CGCCGCCTGCGACGTCGCGAGAAGTCCACTGAACCTTATCATTTAGAGGAAGGAGAAGTCGTAACAAGGT

TTCCGTAGGTGAACCTGCGGAAGGATCATTGTCGAAACCTGCCCAGCAGAACGACCCGCGAACTTGTAGA

GACCACCGGCGGCGGGAGGGGGGATGCGCTCGTCTGCGGGCGCTCCTCCTTCCTCCCCACTCGCCGCTGG

GAGGGGGACTAGTCCCGCTCCTGGCTGGCGAAACAACGAACCCCCGGCGCGGACTGCGCCAAGGAAATCC

AACGAGAGAGCACGCACCCGCGGCCCCGGAGACGGGGTGCCGCGGGGCGCGGCGCCTTCTTTCACTTCTA

TCCAAAATGACTCTCGGCAACGGATATCTCGGCTCTCGCATCGATGAAGAACGTAGCGAAATGCGATACT

TGGTGTGAATTGCAGAATCCCGTGAACCATCGAGTCTTTGAACGCAAGTTGCGCCCTAAGCCGTTAGGCC

GAGGGCACGTCTGCCTGGGTGTCACGCATCGTTGCCCACCCCACCCCCCCCCCATCGAGGCGGGGGCCCG

GAGGAGGTGCGGGCGGAGATTGGCCTCCCGTGCGCGGACCGCTCGCGGTTGGCCCAAATCCGAGTCCTCG

GCGACCGAAGCCGCGGTGATCGGTGGTGAAAAAAAAGCCTCTCGAGCTCCCACCGCACGCCCGGTCTCCA

CGAGGGGACCCTGTGACCCTGACGCCCCGCGCAAGCGGCGGCTCGCATCGCGACCCCAGGTCAGGCGGGA

TCACCCGCTGAGTTTAAGCATATCAATAAGCGGA

*Paramignya scandens* (EF126562.1, EF138912.1),

# Paramignya scandens voucher PI 109758 atpB-rbcL intergenic spacer, partial sequence; and ribulose-1,5-bisphosphate carboxylase/oxygenase large subunit gene, partial cds; chloroplast

GenBank: EF126562.1

[GenBank](https://www.ncbi.nlm.nih.gov/nuccore/EF126562.1?report=genbank) [Graphics](https://www.ncbi.nlm.nih.gov/nuccore/EF126562.1?report=graph) [PopSet](https://www.ncbi.nlm.nih.gov/popset?DbFrom=nuccore&Cmd=Link&LinkName=nuccore_popset&IdsFromResult=123326079)

>EF126562.1 Paramignya scandens voucher PI 109758 atpB-rbcL intergenic spacer, partial sequence; and ribulose-1,5-bisphosphate carboxylase/oxygenase large subunit gene, partial cds; chloroplast

AATTTTTTTTTTTCGAACTTGATCGCATCCAAAAAAATCTTCGATAGCACGTGGATCGGTTAATTCAATA

AGAAATGGGAGTTCGCGCTCGATTTCGTTGGTACCGTCCAACCGAATGCAATTCAATAGTTTCCTTATGC

TTATGCATTTATGCAATTTCAATGAGGGAATTTTCAAGTTCAACCAACCGACTTTCAAAATATCAAGTGG

GTGAATAAAAATCTTGAGAAAGCCATTCATTATATCTATCATTATAGACAATACCCGCCATATTATCTAT

GGAGTTCGAACCTGAACTCTCTTTTTCGATTCATTATTTCTATCTCATTGGACCTTATTTCTTATTTTAG

CATATCGACTTATGCCTAGTCTATTCTTTTTTTTTTATATATATACATATATACCCCGCCTTTCTTTACT

GGATGAATTACGCATATTTTCTTTGCACATATAGGATTTACGTATACAACACATATTACTGTCAAGAGTA

AATTTATTATTATTTAAGATATTTCGATTGAAAAAAGGTAAGGGATTAGAAACTTGAAAGGCGCCGATTG

GGTTGCGCCATACATATGAAAGAGTATACAATAATGATGTATTTGGTGAATCAAATACCATGGTCTAAAA

AAACAAGGAAACGCTCTGAGTAGTTGATAATATTAATTGAAAAGCTTGTGAAAGATTCCTGTGAAGGGGT

TTCATTAACTCCTAATTTATGTCGAGTAGACCTTGTTCTTGCGAGAATTCTTAATTCATGAGTTGTAGGG

AGGGACTTATGTCACCACAAACAGAGACTAAAGCGAGTGTTGGATTCAAGGCCGGTGTTAAAGATTATAA

ATTGACTTATTATACTCCTGACTATGTAACCAAAGATACTGATATCTTGGCAGCAT

# Paramignya scandens isolate SHA0650 maturase K gene, partial cds; chloroplast

GenBank: EF138912.1

[GenBank](https://www.ncbi.nlm.nih.gov/nuccore/EF138912.1?report=genbank) [Graphics](https://www.ncbi.nlm.nih.gov/nuccore/EF138912.1?report=graph) [PopSet](https://www.ncbi.nlm.nih.gov/popset?DbFrom=nuccore&Cmd=Link&LinkName=nuccore_popset&IdsFromResult=126952358)

>EF138912.1 Paramignya scandens isolate SHA0650 maturase K gene, partial cds; chloroplast

GTCGTGTTGTTGAGATCCATCTAGTTCTAAATATACTTGAAATTCCTCCATTTTAAATTCGATTAAAAAC

AAAGGTAAGGGATTTAGTGAGCGATCAAACGATACATAGTGCGATACGGTGAAAACAAAGTATTGTAGTA

AAAAAGGTAGATACCTTGAAAATGGGTAGACTCATCACCGGATTCTCTATCCTCTCATTTCGAGTTAATT

TAATTGGTTTATGTTTGTTATAGTTATAGTATAACTAAGTGGTTAGAAACCCTTTATTTTTTCACTCCAA

TCGCTCTTTTGATTTTGGAAAAAAACAACTATATTTATCAATATACTGCTTCTTCTACACATTCAGCTAC

AACCCATAATAGGGACTCGCTAATACTTAGGACTCATTAAATAAATCGATAATCCCCTCATGGGAAAACC

TTTCCCCGCGTTAGGAACTAATATCTTTTTAACCTTTAATTAGATCGGATAATCATTCAAATTAAGAACC

GAAGCTCGTTACTTTTTGTTTCCCTATAATTGGAACCCTAGGGCTCTATCCATTTATTCACTCGACCGAA

CTCTTAATTAAATTTATTTTGTTCCGCGCCAAGAATTCAAACTTGGTTTTATAGCGATTGAACAAGAATA

AAATATTCTAAAAATTATCCATTGATACGACATGCTGTTTTTTCCATTCATTCCTTTCAGGATCAGTCGC

GGTCTTACAAACTCTCCCGAAGATTTGGACGAATTCTTTGCTTCATAGAAATGTGTAAAAGATGCTAGCC

GACT

*Paramignya monophylla* (AF320881.1)

# Paramignya monophylla atpB/rbcL intergenic spacer, partial sequence; ribulose 1,5-bisphosphate carboxylase gene, partial sequence; chloroplast gene for chloroplast product

GenBank: AF320881.1

[GenBank](https://www.ncbi.nlm.nih.gov/nuccore/AF320881.1?report=genbank) [Graphics](https://www.ncbi.nlm.nih.gov/nuccore/AF320881.1?report=graph)

>AF320881.1 Paramignya monophylla atpB/rbcL intergenic spacer, partial sequence; ribulose 1,5-bisphosphate carboxylase gene, partial sequence; chloroplast gene for chloroplast product

ANACCTAATAAAGTTAAATATGTNAATTTTTTTTTTTTCGAACTTGATCGCATCCAAAAAAATCTTCGAT

AGCACGTGGATCGGTTAATTCAATAAGAAATGGGAGTTCGCGCTCGATTTCGTTGGTACCGTCCAACCGA

ATGCAATTCAATAGTTTCCTTATGCTTATGCATTTATGCAATTTCAATGAGGGAATTTTCAAGTTCAACC

AACCGACTTTCAAAATATCAAGTGGGGTAAATAAAAATTTTGAAAAAGCCGTTCATTATATCTATCATTA

TAGACAATACCCGCCATATTATCTATGGAGTTCGAACCTGAACTCTTTTTTTCGATTCATTATTTCTATC

TCATTGGACCTTATTTCTTATTTTAGCATATCGACTTATGCCTAGTCTATGTCTATTCTTTTTTTTTATA

TATATACCCCGCCTTTTTTTACTGGATGAATTACCGCATATTTTCTTTGCACATATAGGATTTACGTATA

CACACATATTACTGTCAAGAGTCAATTTCTTATTATTTAAGATATTTCGATTGAAAAAAGGTAAGGGATT

AGAAACTTGAAAGGCGCCGATTGGGTTGCGCCATACATATGAAAGAGTATACAATAATGATGTATTTGGT

GAATCAAATACCATGGTCTAAAAAAACAAGGAAACGCTCTGAGTAGTTGATAATATTAATTGAAAAGCTT

GTGAAAGATTCCTGTGAAGGGGTTTCATTAACTCCTAATTTATGTCGAGTAGACCTTGTTCTTGCGAGAA

TTCTTAATTCATGAGTTGTAGGGAGGGACTTATGTCACCACAAACAGAGACTAAAGCGAGTGTTGGATTC

AAGGCCGGTGTTAAAGATTATAAATTGACTTATTATACTCCTGACTATGTAACCAAAGATACTGATATCT

TGGCAGCATTCCGAGTAACTCCTCAACCCGGAGTTCCACCCGAGGAAGCGGGGGCTGCGGTAGCTGCGGA

ATCCTTACTAA

*Paramignya lobata* (EF126561.1, EF138911.1)

# Paramignya lobata voucher PI 600642 atpB-rbcL intergenic spacer, partial sequence; and ribulose-1,5-bisphosphate carboxylase/oxygenase large subunit gene, partial cds; chloroplast

GenBank: EF126561.1

[GenBank](https://www.ncbi.nlm.nih.gov/nuccore/EF126561.1?report=genbank) [Graphics](https://www.ncbi.nlm.nih.gov/nuccore/EF126561.1?report=graph) [PopSet](https://www.ncbi.nlm.nih.gov/popset?DbFrom=nuccore&Cmd=Link&LinkName=nuccore_popset&IdsFromResult=123326077)

>EF126561.1 Paramignya lobata voucher PI 600642 atpB-rbcL intergenic spacer, partial sequence; and ribulose-1,5-bisphosphate carboxylase/oxygenase large subunit gene, partial cds; chloroplast

TTTTTTTTTTTTTCGAACTTGATCGCATCCAAAAAAATCTTCGATAGCACGTGGATCGGTTAATTCAATA

AGAAATGGGAGTTCGCGCTCGATTTCGTTGGTACCGTCCAACCGAATGCAATTCAATAGTTTCCTTATGC

TTATGCATTTATGCAATTTCAATGAGGGAATTTTCAAGTTCAACCAACCAACTTTCAAAATATCAAGTGG

GTGAATAAAAATCTTGAGAAAGCCGTTCATTATATCTATCATTATAGACAATACCCGCCATATTATCTAT

GGAGTTCGAACCTGAACTCTCTTTTTCGATTCATTATTTCTATCTCGTTGGACCTTATTTCTTATTTTAG

CATATCGACTTATGCCTAGTCTATTCTTTTTTTTTATATATATATACCCCGCCTTTCGTTACTGGATGAA

TTACGCATATTTTCTTTGCACATATAGGATTTACGTATACAACACATATTACTGTCAAGAGTCAATTTCT

TATTATTTAAGATATTTCGATTGAAAAAAAGTAAGGGATTAGAAACTTGAAAGGCGCCGATTGGGTTGCG

CCATACATATGAAAGAGTATACAATAATGATGTATTTGGTGAATCAAATACCATGGTCTAAAAAAACAAG

GAAACGCTCTGAGTAGTTGATAATATTAATTGAAAAGCTTGTGAAAGATTCCTGTGAAGGGGTTTCATTA

ACTCCTAATTTATGTCGAGTAGACCTTGTTCTTGCGAGAATTCTTAATTCATGAGTTGTAGGGAGGGACT

TATGTCACCACAAACAGAGACTAAAGCGAGTGTTGGATTCAAGGCCGGTGTTAAAGATTATAAATTGACT

TATTATACTCCTGACTATGTAACCAAAGATACTGATATCTTGGCAGCAT

# Paramignya lobata isolate SHA0652 maturase K gene, partial cds; chloroplast

GenBank: EF138911.1

[GenBank](https://www.ncbi.nlm.nih.gov/nuccore/EF138911.1?report=genbank) [Graphics](https://www.ncbi.nlm.nih.gov/nuccore/EF138911.1?report=graph) [PopSet](https://www.ncbi.nlm.nih.gov/popset?DbFrom=nuccore&Cmd=Link&LinkName=nuccore_popset&IdsFromResult=126952356)

>EF138911.1 Paramignya lobata isolate SHA0652 maturase K gene, partial cds; chloroplast

GTCGTGTTGTTGAGATCTATCTAGTTCTAAATATACTTGAAATTCCTCCATTTGAAATTCGATTAAAAAC

AAAGGTAAGGGATTTAGTGAGCGATCAAACGATACATAGTGCGATACGGTGAAAACAAAGTATTGTAGTA

AAAAAAGTAGATACCTTGAAAATGGGTAGACTCATCACCGGATTCTCTATCCTCTCATTTCGAGTTAATT

TAATTGGTTTATGTTTGTTATAGTTATAGTTATAGTATAACTAAGTGGTTAGAAACCCTTTATTTTTTCA

CTCCAATCGCTCTTTTGATTTTGGAAAAAAACAACTATATTTATCAATATACTGCTTCTTCTACACATTC

AGCTACAACCCATAATAGGGACTCGCTAATACTTAGGACTCATTAAATAAATCGATAATCCCCTCATGGG

AAAACCTTTCCCCGCGTTAGGAACTAATATCTTTTTAACGTTTAATTAGATCGGATAATCATTCAAATTA

AGAACCGAAGCTCGTTACTTTTTGTTTCCCTATAATTGGAACCCTAGGGCTCTATCCATTTATTCACTCG

ACCGAACTCTTAATTAATAATTAAATTTATTTTGTTCCGCGCCAAGAATTCAAACTTGGTTTTATAGCGA

TTGAACAAGAATAAAATATTCTAAAAATTATCCATTGATACGACATGCTGTTTTTTCCATTCATTCCTTT

CAGGATCAGTCGCGGTCTTACAAATATCCCGAAGATTTGGACGAATTCTTTGCTTCATAGAAATGTGAAA

AGATGCTAACCCGTATAT

*Severinia buxifolia* (AB505912.1)

# Severinia buxifolia chloroplast rbcL gene for ribulose-1,5-bisphosphate carboxylase/oxygenase large subunit, partial cds

GenBank: AB505912.1

[GenBank](https://www.ncbi.nlm.nih.gov/nuccore/AB505912.1?report=genbank) [Graphics](https://www.ncbi.nlm.nih.gov/nuccore/AB505912.1?report=graph)

>AB505912.1 Severinia buxifolia chloroplast rbcL gene for ribulose-1,5-bisphosphate carboxylase/oxygenase large subunit, partial cds

AGTATAAGTTGACTTATTATACTCCTGACTATGTAACCAAAGATACTGATATCTTGGCAGCATTCCGAGT

AACTCCTCAGCCCGGAGTTCCACCCGAGGAAGCGGGGGCTGCGGTAGCTGCGGAATCTTCTACTGGTACC

TGGACAACTGTGTGGACCGATGGGCTTACCAGCCTTGATCGTTACAAAGGGCGATGCTACAACATTGAGC

CCGTTGCTGGAGAAGAGAATCAATATATATGTTATGTAGCTTACCCGTTAGACCTTTTTGAAGAAGGTTC

TGTTACTAACATGTTGACTTCCATTGTGGGTAATGTATTTGGTTTCAAAGCACTGCGCGCTCTACGTCTA

GAGGATCTACGAATCCCTCCTGCGTATACTAAAACTTTCCAAGGCCCGCCTCACGGCATCCAAGTTGAGA

GAGATAAATTGAACAAGTATGGACGTCCCCTGTTGGGATGTACTATTAAACCTAAACTGGGGTTATCCGC

TAAGAATTATGGTAGGGCGGTTTATGAATGTCTACGTGGTGGACTTGACTTTACCAAAGATGATGAGAAC

GTGAACTCCCAACCATTTATGCGTTGGAGGGACCGTTTCTTATTTTGTGCGGAAGCACTTTATAAAGCGC

AAGAGGAAACAGGTGAAATCAAAGGTCATTACTTGAATGCTACTGCAGGGACATGCGAAGAAATGATAAA

AAGGGCTGTTTTTGCCAGAGAGTTGGGAGCTCCTATCGTAATGCATGACTACTTAACAGGGGGATTCACC

GCAAATACTAGCTTGGCTCATTATTGCCGAGATAATGGTCTACTTCTTCACATCCACCGTGCAATGCATG

CAGTTATTGATAGACAGAAGAATCATGGTATGCACTTTCGTGTACTAGCTAAAGCTTTGCGTCTGTCAGG

TGGAGATCATATTCACGCCGGTACAGTAGTAGGTAAACTTGAGGGGGAAAGAGACATAACCTTGGGATTT

GTTGATTTACTACGTGATGATTTTGTTGAAAAAGATCGAAGCCGCGGTATTTATTTCACTCAAGATTGGG

TCTCTATACCAGGTGTTATACCTGTGGCTTCCGGGGGTATTCACGTTTGGCATATGCCGGCGTTGACAGA

GATCTTTGGAGATGATGCCGTACTACAATTTGGTGGAGGAACTTTAGGACACCCTTGGGGAAATGCACCC

GGCGCTGTAGCTAATCGAGTAGCTCTAGAAGCATGTGTAAAAGCTCGTAATGAAGGACGCGATCTTGCTC

GCGAAGGTAATGAAATTATCCGGGAGGCTAGCAAATGGAG

**Online Resource 2**

The site for the automatic barcode gap discovery ABGD server has just moved and updated (Last modification date: 06/11/2020 22:56:05s). The new address is: <https://bioinfo.mnhn.fr/abi/public/abgd/>

**
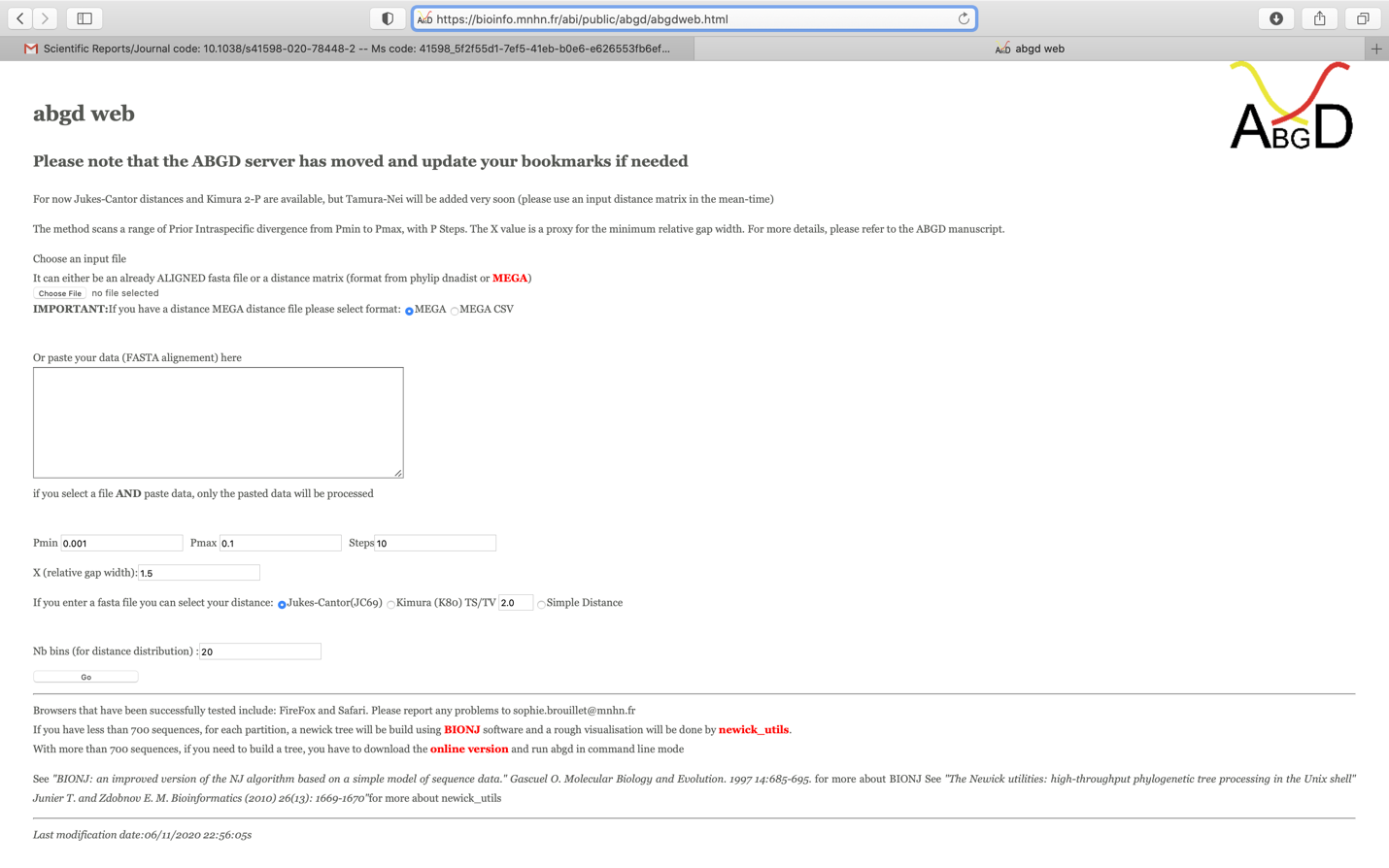
**
